# Outcome measures for children with mitochondrial disease: consensus recommendations for future studies from a Delphi-based international workshop

**DOI:** 10.1007/s10545-018-0229-5

**Published:** 2018-07-19

**Authors:** Saskia Koene, Lara van Bon, Enrico Bertini, Cecilia Jimenez-Moreno, Lianne van der Giessen, Imelda de Groot, Robert McFarland, Sumit Parikh, Shamima Rahman, Michelle Wood, Jiri Zeman, Anjo Janssen, Jan Smeitink

**Affiliations:** 1 Radboud Center for Mitochondrial Medicine, Department of Paediatrics, Radboudumc, Nijmegen, The Netherlands; 2 Unit of Neuromuscular and Neurodegenerative Disorders, Bambino Gesù Children’s Research Hospital, Rome, Italy; 3 Wellcome Centre for Mitochondrial Research, Newcastle University, Newcastle upon Tyne, UK; 4 Center for Lysosomal and Metabolic Diseases and Department of Pediatric Physiotherapy, Erasmus MC—Sophia Children’s Hospital, Rotterdam, The Netherlands; 5 Donders Center for Neuroscience, Department of Rehabilitation, Radboudumc, Nijmegen, The Netherlands; 6 Mitochondrial Medicine Center, Neuroscience Institute, Cleveland Clinic, Cleveland, OH USA; 7 Mitochondrial Research Group, UCL Great Ormond Street Institute of Child Health and Metabolic Unit, Great Ormond Street Hospital NHS Foundation Trust, London, UK; 8 Department of Paediatrics, First Faculty of Medicine and General Faculty Hospital, Prague, Czech Republic; 9 Department of Rehabilitation, Pediatric Physical Therapy, Radboudumc, Nijmegen, The Netherlands

## Abstract

Although there are no effective disease-modifying therapies for mitochondrial diseases, an increasing number of trials are being conducted in this rare disease group. The use of sensitive and valid endpoints is essential to test the effectiveness of potential treatments. There is no consensus on which outcome measures to use in children with mitochondrial disease. The aims of this two-day Delphi-based workshop were to (i) define the protocol for an international, multi-centre natural history study in children with mitochondrial myopathy and (ii) to select appropriate outcome measures for a validation study in children with mitochondrial encephalopathy. We suggest two sets of outcome measures for a natural history study in children with mitochondrial myopathy and for a proposed validation study in children with mitochondrial encephalopathy.

## Introduction

Thirteen researchers from five different countries (seven different mitochondrial medicine centres in the UK, USA, Czech Republic, Italy and the Netherlands) met in Vianen, the Netherlands from 1 to 2 March 2018 to discuss which outcome measures would be best to use in clinical trials for children with mitochondrial myopathy and mitochondrial encephalopathy. The aims of the workshop were to (i) define the protocol for an international, multi-centre natural history study in children with mitochondrial myopathy and (ii) to select appropriate outcome measures for a validation study in children with mitochondrial encephalopathy.

## Background

Mitochondrial diseases are an important group of inherited disorders (Rahman and Rahman 2018), with an estimated prevalence of 1 in 5000 live births (Chinnery and Turnbull 2001; Schaefer et al. 2008; Gorman et al. 2016). The term “mitochondrial diseases”, although in strict sense much broader, is reserved here for disorders directly affecting the function of the oxidative phosphorylation system. Mitochondrial diseases can be caused by mutations in mitochondrial or nuclear DNA (Leonard and Schapira 2000a, b).

Paediatric mitochondrial diseases mostly present with a multi-system phenotype mainly involving tissues with high energy demands, such as the brain, retina, heart, kidney and skeletal muscle (Koopman et al. 2016). For practical reasons, we divided the extremely complex and heterogeneous clinical presentations of individual mitochondrial disease into two phenotypes: mitochondrial encephalopathy, where the central nervous system has the most prominent signs and symptoms, and mitochondrial myopathy, in which the clinical disease expression is dominated by muscle involvement.

Mitochondrial myopathy is a heterogeneous entity with variable severity, aetiology and prognosis. Symptoms in early childhood include feeding problems with poor sucking and, consequently, poor weight gain, hypotonia and delayed motor development. At later age, (proximal) myopathy, exercise intolerance, fatigue, ptosis and external ophthalmoplegia are the most prevalent symptoms. Some patients may be severely limited in their daily activities and fully dependent on wheelchairs and help from caretakers, while others manage to live a reasonably normal life despite their reduced physical capacity (Debray et al. 2007). Some patients with mitochondrial myopathy will develop multi-system disease symptoms later in life, including cardiomyopathy, optic atrophy or sensorineural deafness (de Laat et al. 2012).

The signs and symptoms of patients with mitochondrial encephalopathy are, as with mitochondrial myopathies, highly heterogeneous, ranging from early fatal epilepsy syndromes to episodic migraine at later age. Children typically present with psychomotor retardation and failure to thrive, with or without other neurological features, such as hypotonia, spasticity and epilepsy (Skladal et al. 2003). Depression, behavioural problems and psychosis may complicate the course of the disease (Koene et al. 2009). Brain magnetic resonance imaging (MRI) may show specific or non-specific abnormalities (Valanne et al. 1998), although many have no visible abnormalities on MRI. This highly heterogeneous group of patients mostly shows a variable intellectual disability, which frequently requires the patient to attend a special school and/or remain dependent on caretakers (Debray et al. 2007). Also, in mitochondrial encephalopathy, multiple organ systems may be involved.

While an increasing number of clinical trials is emerging for mitochondrial disease, there is a paucity of effective therapies (Gorman et al. 2016; Koopman et al. 2016; Viscomi 2016). To define the effectiveness of any new pharmacologic agent, well-designed clinical studies need to be executed. Previously, a Cochrane review on treatment studies in patients with mitochondrial disease stated that the quality of many of the previously performed studies is poor (Pfeffer et al. 2013). One of the main problems arising in these low-quality studies includes the heterogeneity of the often small-numbered study population and the lack of sufficiently sensitive patient-centred outcome measures.

To prepare for clinical trials in children with mitochondrial diseases, the phenotype and natural disease course of children with mitochondrial disease should be well known (Keshavan 2018). Only if the complaints, symptoms and functional abilities and their variability or progression over time are clear can appropriate outcome measures be selected.

Based on the knowledge of the clinical spectrum of the disease, the disease course and the functional abilities of the patients, outcome measures can be selected. Selection of an outcome measure should take into account the final aim of using it, namely to detect a clinically relevant difference between the active and the placebo group. The regulatory agencies [e.g. the European Medicines Agency (EMA) and the U.S. Food and Drug Administration (FDA)] prefer the direct measurement of “how a patient feels, functions or survives”, using functional outcome measures and patient-reported outcome measures (FDA 2009). A recent study evaluating the effectiveness of orphan medicinal products in the real world deemed that the use of a clinical or validated surrogate primary endpoint seemed to be related to effectiveness in the real world (Schuller et al. 2017). This stresses the importance of not only selecting a clinically relevant endpoint but also the accurate validation of surrogate endpoints. Surrogate endpoints can only substitute for a clinical endpoint when there is confirmation of a strong relation with the pathophysiology of the disease, clinical response to therapy and the prediction of clinical benefit (Schuller et al. 2017). Although validated surrogate endpoints are suitable for early-stage clinical trials, clinical and functional endpoints are the closest reflection of patients’ functioning and should be used in later stages of drug development (Cox 2018).

There have been several initiatives to harmonise clinical and functional outcome measures for children with mitochondrial disease. In 2013, some of our group published a review on which outcome measures can be used for children with mitochondrial diseases, based on the experience in other diseases (Koene et al. 2013). Nearly 4 years later, a group of researchers gathered in Rome, Italy to identify outcome measures for children and adults with mitochondrial myopathy (Mancuso et al. 2017). As a follow-up to the workshop in Rome, this workshop aimed to establish consensus on which outcome measures to use in natural history studies in children with mitochondrial myopathy, based on experience with these outcome measures in a validation study and in daily practice. The aim of the second day was to identify outcome measures to be investigated in a validation study in children with mitochondrial encephalopathy.

## Methods

### Delphi-based method

The Delphi method provides a systematic approach to collect opinions from experts (the “Delphi panel”) and has been used to obtain consensus or to provide recommendations on a well-defined and specified topic (Jorm 2015). In this method, experts provide their opinions anonymously, on an individual and independent basis. In our case, we have used several elements of this method to guide our workshop (therefore, called Delphi-based method). We brought a wide range of international experts in mitochondrial medicine and neuromuscular disease with diverse professional backgrounds together. The experts were provided with background information and discussions before they expressed their opinions. Moreover, a second voting round was executed after presentation and discussion of the results in the first voting round, allowing an iterative process.

### Pre-meeting

Experts from established centres of excellence in the diagnosis and management of children with mitochondrial disease were invited for a two-day workshop on outcome measures in paediatric mitochondrial disease. The Delphi panel consisted of three metabolic paediatricians, three paediatric neurologists, one paediatric rehabilitation specialist, one clinical pharmacologist with a special interest in trial design in mitochondrial disease and five paediatric physiotherapists with a broad clinical background. Participants were asked to give their input for functional outcome measures they thought were suitable to measure disease progression in a natural history study, based on their clinical experience. All participants were asked to prepare a short introduction about two to four of the outcome measures in the list and the measurement properties in patients with mitochondrial disease and—when not available—in other diseases.

### Meeting

Both days started with a presentation on mitochondrial myopathy and mitochondrial encephalopathy, respectively, where the clinical spectrum of the disease was illustrated by one of the experienced physicians. Subsequently, all participants presented one or more outcome measures, including the measurement protocol, previously published evidence on the psychometric and clinimetric properties of the tool, previous experience in mitochondrial disease (and in other similar diseases with the respective measure), results from their own clinical studies as well as pros and cons of using a particular outcome measure in future studies.

After all the outcome measures had been presented, an online survey was completed, designed to obtain the level of consensus regarding inclusion and exclusion criteria, definitions and the use of these specific outcome measures. First, aspects of protocol design, including the time between measurements and the inclusion and exclusion criteria, were voted for online. We predefined cut-offs in advance, namely: a “strong consensus” for a statement was considered to have been reached when more than 90% of scores were positive or negative (“yes” or “no”), “good consensus” was defined as more than 70% of the participants voting in the same direction. For the outcome measures, participants voted using a 5-point Likert scale to indicate their level of agreement on each statement that this test should be included as an instrument in the natural history study (1 = absolutely disagree, 2 = disagree, 3 = no judgment, 4 = more than agree, 5 = absolutely agree). The pre-defined “strong consensus” for a statement was considered to have been reached when both more than 70% of scores were ≥ 4 and the mean score was > 4. In instances where an expert did not have an opinion on one of the items, (s)he was asked to leave the item open (so this item would not be scored for this expert). If only one of these two parameters were met, this was considered a “good consensus”. If both parameters were not met in either the positive or negative directions, then the statement was considered to lack consensus agreement (Mancuso et al. 2017). The questionnaire also contained a checkbox in which participants were asked to select their top 5 priority outcome measures for future studies. The results were analysed anonymously. There were two voting rounds (after the presentations of the outcome measures and after presenting and discussing the results of the first round) for both mitochondrial myopathy and for mitochondrial encephalo(myo)pathy.

#### Data availability

Not applicable.

## Workshop

The aim of the first day was to design a natural history protocol for children with mitochondrial myopathy, with a strong focus on which outcome measures to use. We adopted the definition of primary mitochondrial myopathy (PMM) from the PMM working group (Mancuso et al. 2017), namely that “mitochondrial myopathies are genetically defined disorders leading to defects of oxidative phosphorylation affecting predominantly, but not exclusively, skeletal muscle”.

The first day started with a presentation about the clinical spectrum of mitochondrial myopathy in children, illustrating the complex phenotypes of childhood mitochondrial myopathy. The most important complaints include proximal myopathy, fatigue, exercise intolerance, muscle pain, ophthalmoplegia, respiratory muscle weakness and, rarely, rhabdomyolysis. Pure myopathy as the sole clinical disease expression in children with mitochondrial disease is extremely rare.

Subsequently, the design and results of a recent study exploring outcome measures in children with mitochondrial myopathy (registration number NL59491.091.16) were presented. Experience (including a literature review) with instruments selected by the PMM working group which were not covered by this study were also presented by (other) members of the Delphi panel.

On the second day, outcome measures for a validation study in children with mitochondrial encephalo(myo)pathy were selected. First, the wide clinical spectrum of mitochondrial encephalopathy, ranging from early fatal epilepsy syndromes to the later onset mitochondrial encephalopathy with lactic acidosis and stroke-like episodes (also known as MELAS syndrome), was illustrated. Subsequently, a literature review of and experience with the proposed instruments in mitochondrial encephalo(myo)pathy were presented.

After a discussion on the domains to be covered, the feasibility, reliability, validity and responsiveness of the tests presented and how to integrate these in a natural history protocol, all participants voted on which tests should be included. These results were subsequently discussed and items on which no consensus was reached (either more than 70% of scores were ≥ 4 or the mean score was > 4 but not both) were discussed again.

## Results of the Delphi-based process

### Mitochondrial myopathy

The inclusion criteria for the natural history study include all children with mitochondrial myopathy who are 0–18 years and have a pathogenic mutation related to mitochondrial disease. Asymptomatic carriers may also be included. An exclusion criterion is doubt about the pathogenicity of the mutation. The time between measurements was agreed as 6 months. It was agreed that, in rapidly progressive and young patients (< 4 years), the time between measurements should be decreased to 3 months. Motor function, endurance and muscle power were identified as the most important symptoms to be covered by selected outcome measures. Only tests with more than 70% of the raters agreeing to include the test and/or an average rating ≥ 4.0 were included in Tables 1 and 2. Outcome measures were prioritised (Table 2) and the sequence of the tests was determined for the natural history protocol (available upon request).

**Table 1:** Results of the Delphi-based process

	Consensus	
	Percentage scores ≥ 4	Mean score
Myopathy		
6-min walk test	100%	4.4
30 s sit to stand test	100%	4.4
Children’s Hospital of Philadelphia Infant Test of Neuromuscular Disorders (CHOP-INTEND)	100%	4.7
Growth and weight gain	100%	4.6
Caregiver burden scale	100%	4.7
Grip strength	92%	4.2
New muscle endurance test (developed by RCMM)	92%	4.0
International Paediatric Mitochondrial Disease Scale (IPMDS)	91%	4.3
PedsQL (multidimensional fatigue)	91%	4.2
Newcastle Paediatric Mitochondrial Disease Scale (NPMDS)	82%	4.0
Timed tests (stand up from floor)	77%	3.8
* Hammersmith Functional Motor Scale*	64%	3.6
* Accelerometer*	62%	3.5
* Alberta Infant Motor Scale (AIMS)*	60%	3.6
*Gait measurement (GAITRite)*	55%	3.4
* PedsQL (general)*	54%	3.5
* Attain stand test (30 s)*	46%	2.9
* Timed tests (10-m walk)*	46%	3.3
* North Star Ambulatory Assessment*	44%	3.2
*Test of Masticating and Swallowing Solids (TOMASS)*	40%	3.2
* Checklist individual strength (CIS)*	38%	3.1
* PedsQL (neuromuscular)*	36%	3.2
* Lateral step-up test (30 s)*	31%	2.6
*Childhood Myositis Assessment Scale (CMAS)*	31%	2.8
*Gross Motor Function Measure (GMFM)*	27%	2.7
*Pediatric Evaluation of Disability Inventory (PEDI)*	23%	2.6
* Eyelid ptosis and ophthalmoparesis tests*	18%	2.3
* 9-hole peg test*	15%	1.8
* Quantitative muscle power using hand-held dynamometer*	15%	2.3
*Timed tests (5 stairs)*	15%	2.2
* Quantitative myasthenia gravis test*	8%	2.0
* 6-min mastication test*	0%	1.8
Encephalo(myo)pathy		
Gross Motor Function Measure (GMFM)	100%	4.7
Pediatric Evaluation of Disability Inventory (PEDI)	100%	4.4
Spasticity: Tardieu scale	100%	4.2
Caregiver burden scale	100%	4.5
Barry–Albright Dystonia Scale	92%	4.1
Scale for the assessment and rating of ataxia (SARA)	85%	4.1
Growth and weight gain	85%	4.2
Alberta Infant Motor Scale (AIMS)	85%	3.9
Children’s Hospital of Philadelphia Infant Test of Neuromuscular Disorders (CHOP-INTEND)	85%	4.0
6-min walk test	85%	3.9
9-hole peg test	85%	3.8
Timed tests (10-m walk)	85%	3.8
Pediatric Outcomes Data Collection Instrument (PODCI)	77%	3.7
30 s sit to stand test	77%	3.8
International Pediatric Mitochondrial Disease Scale (IPMDS)	73%	4.1
*CP classifications (CFCF, GMFCS, MACS)*	69%	3.8
*Global assessment of three most important symptoms*	67%	3.8
* Accelerometer*	54%	3.5
* Timed tests (stand up from floor)*	46%	3.1
* Perceive Recall Plan Perform (PRPP)*	38%	2.8
* Peabody vocabulary test*	38%	3.2
* PedsQL (general)*	33%	3.1
*Test of Masticating and Swallowing Solids (TOMASS)*	31%	3.0
* Gait measurement (GAITRite)*	31%	3.0
* Timed tests (up and go)*	31%	3.0
* Hypertonia Assessment Scale*	23%	2.8
* Child Health Assessment Questionnaire (CHAQ)*	23%	2.8
* KIDSCREEN*	23%	2.7
* Frontal assessment battery*	23%	2.9
* PedsQL (CP)*	15%	2.7
*Luria*	15%	2.8
* Spasticity: Ashworth scale*	8%	2.6
* Timed tests (5 stairs)*	8%	2.5
* Eyberg Child Behavior Inventory*	8%	1.9
* Gross Motor Function Measure (GMFM)*	0%	2.2

The items on which no strong consensus was reached are shown in *italics*

**Table 2:** Proposed outcome measures for the natural history study in children with mitochondrial myopathy

Test	≥ 4 (%)	Mean	Priority adult-like	Priority infantile
6-min walking test	100%	4.4	46%	
30 s sit to stand test	100%	4.4	31%	
Children’s Hospital of Philadelphia Infant Test of Neuromuscular Disorders (CHOP-INTEND)	100%	4.7		92%
Growth and weight gain	100%	4.6	92%	92%
Caregiver burden scale	100%	4.7	92%	100%
Grip strength	92%	4.2	46%	
New muscle endurance test (developed by RCMM)	92%	4	31%	
International Paediatric Mitochondrial Disease Scale (IPMDS)	91%	4.3	54%	54%
PedsQL (multidimensional fatigue)	91%	4.2	46%	
Newcastle Paediatric Mitochondrial Disease Scale (NPMDS)	82%	4	23%	31%
*Timed tests (stand up from floor)*	77%	3.8	11%	

The item on which only good consensus was obtained is shown in *italics*

### Mitochondrial encephalo(myo)pathy

For the outcome measure validation study in children with mitochondrial encephalo(myo)pathy, the time between the training session for the child and the baseline measurement was set as 2 weeks and the time between baseline and re-test was set to be 1 week. At baseline, the child will be measured by two independent raters to test intra-rater reliability; all suitable measurements will be videotaped and scored 6 weeks later to test inter-rater reliability. The results of the baseline and the outcome will be compared to test the in-time variability over time (test–retest reliability). Motor capacity, tone (spasticity, dystonia), ataxia, cognition, quality of life and caregiver burden were identified as the most important domains to be assessed in children with encephalopathy. Because of the wide range in age and functional capacities, more than one outcome measure were selected to be tested for each domain. For example, the Alberta Infant Motor Scale (AIMS) was selected for infants or young children with very low motor capacities, while the other end of the spectrum is covered by the 6-min walking test. Only the tests with either more than 70% of the raters agreeing to include the test or an average rating ≥ 4.0 are included (Tables 1 and 3).

**Table 3:** Proposed outcome measures for the explorative study in children with mitochondrial encephalo(myo)pathy

Test	≥ 4 (%)	Mean	Priority
Gross Motor Function Measure (GMFM)	100%	4.7	100%
Pediatric Evaluation of Disabilities Inventory (PEDI-CAT)	100%	4.4	69%
Tardieu test for spasticity	100%	4.2	77%
Caregiver burden scale	100%	4.5	100%
Barry–Albright Dystonia Scale	92%	4.1	54%
*6-min walk test*	85%	3.9	54%
*9-hole peg test*	85%	3.8	23%
*10-m walk or run test*	85%	3.8	38%
Scale for the Assessment and Rating of Ataxia (SARA)	85%	4.1	54%
Growth and weight gain	85%	4.2	54%
*Alberta Infant Motor Skills (AIMS; for young children)*	85%	3.9	62%
Children’s Hospital of Philadelphia Infant Test of Neuromuscular Disorders (CHOP-INTEND; for young children)	85%	4.0	62%
Newcastle Paediatric Mitochondrial Disease Scale (NPMDS)	85%	4.0	77%
*30 s sit to stand test*	77%	3.8	38%
*Pediatric Outcomes Data Collection Instrument (PODCI)*	77%	3.7	54%
International Pediatric Mitochondrial Disease Scale (IPMDS)	78%	4.0	62%

The items on which only good consensus was obtained are shown in *italics*

## Discussion

The aim of the natural history study is to monitor the motor function, endurance, activities of daily life and muscle power of children with mitochondrial myopathy. Special attention will be paid to standardisation, to not only improve the quality of the data obtained by this multi-centre study, but also to avoid diurnal variation in e.g. ptosis, muscle strength and attention span. Some critical points in these standardisation procedures were discussed during the workshop and will be implicated in the standard operating procedures of the natural history protocol. We have selected a broad pallet of outcome measures, because it is not clear yet on which clinical signs and symptoms future clinical trials will focus. However, it was clear that there should be a balance between the burden to patients and parents and the broadness of information required. By sequencing the assessments based on the established priority ranking, we would also allow centres with limited resources to gather a minimum set of meaningful information in a standardised manner. Since some tests, such as the 6-min walk test, are subject to training effects (Casey et al. 2012), a training session is required in patients who have not recently performed this test.

The aim of the validation study in children with mitochondrial encephalo(myo)pathy is to obtain experience with the selected instruments in affected patients. Based on the results of this validation study, the inclusion and exclusion criteria and the outcome measures for future natural history studies in this population will be discussed in a future workshop. At this point, it was agreed that, because of the lack of sufficient expertise in the present panel, no outcome measures for cognition were selected. It is recommended that these measures are discussed in a separate workshop to harmonise the instruments for this important aspect of brain-related mitochondrial diseases.

The scientific rigour of expert consensus is fully dependent on the evidence this consensus is based upon (Jorm 2015). In this workshop, experts were able to base their opinion on a thorough review of the experience with this outcome measure in mitochondrial and non-mitochondrial diseases by one of the other members of the Delphi panel, as well as the results of a small validation study in four children with mitochondrial myopathy. For paediatric mitochondrial encephalopathy, the opinions were based on a thorough review of the experience with this outcome measure in mainly non-mitochondrial diseases. Therefore, the selected outcome measures will be tested in a validation study in which more evidence for mitochondrial diseases will be generated. Although our workshop process deviated significantly from an optimum Delphi process, including the standardisation of literature review and formulation of questions, the diverse educational and medical background and geographic localisation of the experts contributing to this workshop, as well as the independent decisions of the experts during voting, were compliant with the Delphi method (Jorm 2015).

## Conclusion

In this two-day key opinion leader workshop on outcome measures for children with mitochondrial diseases, we proposed a set of outcome measures for a natural history study in children with mitochondrial myopathy (Table 2), as well as tests for children with mitochondrial encephalopathy (Table 3). The latter tests will be included in a validation study in Nijmegen, to study the feasibility, test–retest reliability and inter- and intra-rater reliability in children with mitochondrial encephalopathy.

## References

[CR1] Casey AF, Wang X, Osterling K (2012). Test–retest reliability of the 6-minute walk test in individuals with Down syndrome. Arch Phys Med Rehabil.

[CR2] Chinnery PF, Turnbull DM (2001). Epidemiology and treatment of mitochondrial disorders. Am J Med Genet.

[CR3] Cox GF (2018). The art and science of choosing efficacy endpoints for rare disease clinical trials. Am J Med Genet A.

[CR4] de Laat P, Koene S, van den Heuvel LP, Rodenburg RJ, Janssen MC, Smeitink JA (2012). Clinical features and heteroplasmy in blood, urine and saliva in 34 Dutch families carrying the m.3243A > G mutation. J Inherit Metab Dis.

[CR5] Debray FG, Lambert M, Chevalier I (2007). Long-term outcome and clinical spectrum of 73 pediatric patients with mitochondrial diseases. Pediatrics.

[CR6] Food and Drug Administration (2009) Guidance for industry: patient-reported outcome measures: use in medical product development to support labeling claims. U.S. Department of Health and Human Services 10.1186/1477-7525-4-79 PMC1629006 17034633

[CR7] Gorman GS, Chinnery PF, DiMauro S (2016). Mitochondrial diseases. Nat Rev Dis Primers.

[CR8] Jorm AF (2015). Using the Delphi expert consensus method in mental health research. Aust N Z J Psychiatry.

[CR9] Keshavan NRS (2018) Natural history of mitochondrial disorders: a systematic review. Essays Biochem (in press) 10.1042/EBC20170108 29980629

[CR10] Koene S, Kozicz TL, Rodenburg RJ (2009). Major depression in adolescent children consecutively diagnosed with mitochondrial disorder. J Affect Disord.

[CR11] Koene S, Jansen M, Verhaak CM, De Vrueh RL, De Groot IJ, Smeitink JA (2013). Towards the harmonization of outcome measures in children with mitochondrial disorders. Dev Med Child Neurol.

[CR12] Koopman WJ, Beyrath J, Fung CW (2016). Mitochondrial disorders in children: toward development of small-molecule treatment strategies. EMBO Mol Med.

[CR13] Leonard JV, Schapira AH (2000). Mitochondrial respiratory chain disorders I: mitochondrial DNA defects. Lancet.

[CR14] Leonard JV, Schapira AH (2000). Mitochondrial respiratory chain disorders II: neurodegenerative disorders and nuclear gene defects. Lancet.

[CR15] Mancuso M, McFarland R, Klopstock T, Hirano M, consortium on Trial Readiness in Mitochondrial Myopathies (2017). International workshop:: outcome measures and clinical trial readiness in primary mitochondrial myopathies in children and adults. Consensus recommendations. 16–18 November 2016, Rome, Italy. Neuromuscul Disord.

[CR16] Pfeffer G, Horvath R, Klopstock T (2013). New treatments for mitochondrial disease-no time to drop our standards. Nat Rev Neurol.

[CR17] Rahman J, Rahman S (2018) Mitochondrial medicine in the omics era. Lancet. pii: S0140-6736(18)30727-X 10.1016/S0140-6736(18)30727-X 29903433

[CR18] Schaefer AM, McFarland R, Blakely EL (2008). Prevalence of mitochondrial DNA disease in adults. Ann Neurol.

[CR19] Schuller Y, Hollak CEM, Gispen-de Wied CC, Stoyanova-Beninska V, Biegstraaten M (2017). Factors contributing to the efficacy–effectiveness gap in the case of orphan drugs for metabolic diseases. Drugs.

[CR20] Skladal D, Sudmeier C, Konstantopoulou V (2003). The clinical spectrum of mitochondrial disease in 75 pediatric patients. Clin Pediatr (Phila).

[CR21] Valanne L, Ketonen L, Majander A, Suomalainen A, Pihko H (1998). Neuroradiologic findings in children with mitochondrial disorders. AJNR Am J Neuroradiol.

[CR22] Viscomi C (2016). Toward a therapy for mitochondrial disease. Biochem Soc Trans.

